# Specification, validation, and adherence of quality indicators to optimize the safe use of nonsteroidal anti-inflammatory drugs for knee osteoarthritis pain in the primary care setting

**DOI:** 10.1186/s12891-023-06904-x

**Published:** 2023-09-27

**Authors:** Joshua T. Swan, Elsie Rizk, Phuong Y Duong, Bader M. Alghamdi, Navjot Kaur, Sudha Nagaraj, Anthony E. Brown, Eleazar Flores, Nathan Spence, Sharla Tajchman

**Affiliations:** 1 Department of Pharmacy, Houston Methodist, Houston, TX USA; 2 Department of Surgery, Houston Methodist, Houston, TX USA; 3 Center for Outcomes Research, Houston Methodist, Houston, TX USA; 4 Houston Methodist Primary Care Group, Houston Methodist, Houston, TX USA; 5 Pfizer, Inc, New York, NY USA

**Keywords:** Osteoarthritis, Pain, Quality indicators, Primary care, NSAIDs

## Abstract

**Background:**

Nonsteroidal anti-inflammatory drugs (NSAIDs) used for osteoarthritis (OA) in primary care may cause gastrointestinal or renal injury. This study estimated adherence to two quality indicators (QIs) to optimize NSAID safety: add proton pump inhibitors (PPI) to NSAIDs for patients with gastrointestinal (GI) risk (QI #1 NSAID-PPI) and avoid oral NSAIDs in chronic kidney disease (CKD) stage G4 or G5 (QI #2 NSAID-CKD).

**Methods:**

This retrospective study included index primary care clinic visits for knee OA at our health system in 2019. The validation cohort consisted of a random sample of 60 patients. The remainder were included in the expanded cohort. Analysis of structured data extracts was validated against chart review of clinic visit notes (validation cohort) and estimated QI adherence (expanded cohort).

**Results:**

Among 60 patients in the validation cohort, analysis of data extracts was validated against chart review for QI #1 NSAID-PPI (100% sensitivity and 91% specificity) and QI #2 NSAID-CKD (100% accuracy). Among 335 patients in the expanded cohort, 44% used NSAIDs, 27% used PPIs, 73% had elevated GI risk, and only 2% had CKD stage 4 or 5. Twenty-one percent used NSAIDs and had elevated GI risk but were not using PPIs. Therefore, adherence to QI #1 NSAID-PPI was 79% (95% CI, 74–83%). No patients with CKD stage 4 or 5 used NSAIDs. Therefore, adherence to QI #2 NSAID-CKD was 100%.

**Conclusion:**

A substantial proportion of knee OA patients with GI risk factors did not receive PPI with NSAID therapy during primary care visits.

**Supplementary Information:**

The online version contains supplementary material available at 10.1186/s12891-023-06904-x.

## Background

Osteoarthritis (OA) pain impairs activities of daily living for over 30 million adults in the United States and accounts for over $370 billion in annual direct medical costs [1–4]. Although numerous guidelines provide evidence-based recommendations to optimize OA pain management, primary care practice may not correlate with guideline recommendations [5–8]. To bridge guideline recommendations to practice, a multi-disciplinary expert panel recently engaged in a consensus strategy that was informed by literature to develop prioritized, valid, and feasible quality indicators (QIs) that can be used to track quality initiatives for OA pain management in the primary care setting [9]. The expert panel determined that the top two priority QIs focused on optimizing safe use of nonsteroidal anti-inflammatory drugs (NSAIDs): add proton pump inhibitors (PPI) to NSAIDs for patients with gastrointestinal (GI) risk (QI #1 NSAID-PPI) [10–14] and avoid oral NSAIDs in chronic kidney disease (CKD) stage G4 or G5 (QI #2 NSAID-CKD) [10]. Among patients on NSAIDs with GI risk, co-administration of PPIs can decrease risk of upper GI complications such as ulceration, perforation, obstruction, or bleeding [15, 16]. The use of NSAIDs in patients with CKD can cause acute kidney injury and a more rapid progression to end-stage renal disease [17].

Additional research is needed to assist clinicians and scientists with evaluation of these new QIs. An operational definition—specific method for calculating success or failure—is needed for each new QI. Large scale programs for quality surveillance and intervention will rely on evaluation of structured healthcare data, and the quality of the necessary structured data elements should be validated against a reference standard, such as chart review of progress notes and medication lists. Once an operational definition has been sufficiently validated, the adherence of the QI should be measured in a population to identify the magnitude of concern and to identify meaningful opportunities to improve care. To directly address these gaps in knowledge, the objective of this study was to (i) specify operational definitions, (ii) validate data extraction against chart review, and (iii) evaluate adherence for two high-priority QIs for OA pain management that were related to NSAID utilization [9].

## Methods

### Setting and study sample

This retrospective study was conducted at the Houston Methodist health system which consists of 148 primary care physicians (PCPs) who practice at 39 locations in the greater urban area of Houston, Texas [18]. All locations utilize Epic (Epic Systems Cooperation, Verona, WI, USA) as their shared electronic health record (EHR). We included all primary care clinic visits at our health system in 2019 for knee OA, defined as a principal international classification of diseases, tenth revision, clinical modification (ICD-10-CM) diagnosis code of M17.0 bilateral primary osteoarthritis of knee; M17.11 unilateral primary osteoarthritis, right knee; M17.12 unilateral primary osteoarthritis, left knee; or M17.10 unilateral primary osteoarthritis, unspecified knee. If a patient had more than one eligible primary care clinic visit in 2019, only the first (index) visit was included in this study. A common denominator was used for both QIs to allow for simultaneous comparison and to determine which QI impacts the largest group of patients.

### Design

This study specified, validated, and estimated adherence for 2 QIs for safe use of NSAIDs. First, QI operational definitions were developed based on literature review and feasibility assessment of existing data elements in the EHR. Second, a data extract was validated against chart review in the validation cohort, consisting of a simple random sample of 60 patients. The health system’s information technology department provided data extracts of ICD-10-CM diagnosis codes (problem lists and active diagnoses), medication lists, serum creatinine values, and estimated glomerular filtration rates (eGFR) values. For the reference standard of chart review, residency-trained clinical pharmacists abstracted medical history, medication use, and kidney function from clinic visit notes and patient communications in the EHR using standardized data collection forms and procedures. Adherence to QIs was evaluated using data extracts and compared to adherence derived from the reference standard chart review. Third, QI adherence was evaluated in an expanded cohort of patients with primary care visits for knee OA who were not otherwise included in the validation cohort. The Houston Methodist Research Institute’s Institutional Review Board approved this study with a waiver of informed consent.

### Operational definition of GI risk

Although several OA guidelines recommend the use of cyclooxygenase-2 selective inhibitors or concurrent PPI to reduce GI injury from NSAIDs among patients with elevated GI risk, these guidelines do not provide operational definitions for GI risk [10–14]. Most proposed GI risk criteria include a history of peptic ulcer disease (PUD) or concomitant aspirin, antiplatelet therapy, anticoagulant therapy, or systemic steroids [15–17, 19–22]. Although advanced age is generally regarded as a risk factor, age thresholds of > 60, >65, > 70, or > 75 years have been proposed [15–17, 20–22]. Some GI risk criteria include the use of serotonin reuptake inhibitors [22] or state that a history of *Helicobacter pylori* infection should be given special consideration [15, 16, 19, 22].

The 2009 American College of Gastroenterology criteria for high risk or moderate risk were used to define GI risk for analysis of QI #1 NSAID-PPI in our study [15]. The 2009 American College of Gastroenterology guidelines provide a pragmatic definition that can be operationalized using structured EHR data as the presence of any of the following risk factors: age > 65, history of PUD (complicated or uncomplicated), or concomitant use of aspirin (including low dose), antiplatelets, anticoagulants, or steroids [15]. Peptic ulcer disease was defined in this study as ulcers of the esophagus, stomach, or duodenum. Ulcers in the jejunum, ileum, or large intestine were considered lower-GI ulcers and did not meet the definition of PUD for this study. During chart review, any mention of an ulcer in the esophagus, stomach, or duodenum or a history of GI bleeding related to NSAID therapy was abstracted as a history of PUD. For analysis of the data extract, the presence of an active ICD-10-CM diagnosis code in the EHR problem list or EHR active diagnoses during the clinic visit was used to identify a history of PUD (Supplemental Table 1 of Additional File 1).

### Operational definition of CKD

Use of NSAIDs can lead to acute kidney injury (AKI). Underlying CKD exacerbates the risk of NSAID-related AKI, which then leads to a more rapid progression of CKD towards end stage renal disease [23, 24]. Current international Kidney Disease: Improving Global Outcomes (KDIGO) Clinical Practice Guidelines define CKD as eGFR < 60 mL/min/1.73 m^2^ with stages based on eGFR: stage G3 as eGFR of 30–59 mL/min/1.73 m^2^, stage G4 as eGFR 15–29 mL/min/1.73 m^2^, and stage G5 as eGFR < 15 mL/min/1.73 m^2^ [25]. These guidelines recommend using the 2009 Chronic Kidney Disease Epidemiology Collaboration (CKD-EPI) equation to calculate eGFR using serum creatinine, age, sex, and African American race [25, 26]. Although there is uncertainty on whether strict avoidance of NSAIDs is necessary for CKD stage G3 based on comorbid conditions and concomitant medications, several guidelines recommend strict avoidance of NSAIDs for OA pain among patients with CKD stages G4 and G5 (eGFR < 30 mL/min/1.73 m^2^) [17, 23, 25, 27–29].

We defined CKD severity that is a contraindication for NSAID therapy as KDIGO stage G4-G5 (eGFR < 30 mL/min/1.73 m^2^) using the CKD-EPI equation that is adjusted for African American race for analysis of QI #2 NSAID-CKD in our study. During chart review, any mention of CKD stages G4 or G5 in the clinic visit notes was abstracted as CKD. For analysis of the data extract, the presence of an active ICD-10-CM diagnosis code for CKD stage G4 or G5 in the EHR problem list or EHR active diagnoses during the clinic visit was used to identify CKD as well as the presence of any eGFR < 30 mL/min/1.73 m^2^ in the EHR in the 12 months preceding the clinic visit (Supplemental Table 2 of Additional File 1). Our health system’s EHR automatically calculates eGFR using the CKD-EPI equation for each serum creatinine value.

### Operational definition of concomitant medication use

For each encounter, the EHR provides a structured medication list that includes prescription medications and over the counter (OTC) medications. This list can be updated by the patient during electronic check in prior to the appointment and is routinely updated by a medical assistant and reconciled by the PCP during each primary care visit. For this study, concomitant medication use was defined as the presence of a medication on the structured medication list as an active home medication, newly prescribed medication during the visit, or new prescription that was entered into the EHR a few days after the visit as part of same EHR encounter. Home medications that were discontinued or removed from this medication list during the primary care visit were not considered concomitant medications. For analysis of the data extract, a list of these structured medications that were active/new and not discontinued during the encounter was provided. For chart review, clinic visit notes and patient communications in the EHR were also reviewed in addition to this medication list. Residency-trained pharmacist investigators categorized every active medication as NSAID (including selective and non-selective), PPI, antiplatelet agent, anticoagulant, aspirin, or systemic steroid. Topical NSAIDs were not included. Short, tapered courses of oral steroids or intramuscular steroid injections were not included.

#### Operational definition of quality indicators

For each QI, patient encounters were categorized as adherent (1 point) or non-adherent (0 point). For QI #1 NSAID-PPI of “add a PPI if oral NSAIDs are used in patients with elevated GI risk,” patients were categorized as follows: no NSAID or NSAID discontinued (1 point), NSAID but no GI risk (1 point), NSAID and GI risk with PPI (1 point), or NSAID with GI risk but no PPI (0 point). For QI #2 NSAID-CKD of “avoid oral NSAIDs among patients with CKD stage G4 or G5,” patients were categorized as follows: No NSAID or NSAID discontinued (1 point), NSAID but no CKD stage G4 or G5 (1 point), or NSAID with CKD stage G4 or G5 (0 point).

### Data management and analysis

Performance characteristics of accuracy, sensitivity, and specificity were calculated for analysis of data extracts compared with the reference standard of chart review. Each patient was coded as being positive or negative for a characteristic of interest (disease presence, medication use, or QI adherence) using both chart review and data extract. Among patients positive via chart review (true positive), sensitivity was calculated as the proportion who were also positive via data extract (screen positive). Among patients negative via chart review (true negative), specificity was calculated as the proportion who were also negative via data extract (screen negative). Accuracy was calculated as number of patients with agreement between approaches (both positive or both negative) divided by the total number of patients evaluated. Abstracted data from chart review was managed using Research Electronic Data Capture tools [30]. Demographics were compared between validation and expanded cohorts using the Chi-square test and Student’s t-test. All statistical analyses were performed using STATA (version 16, StataCorp LLC, College Station, Texas, United States).

## Results

Our study included 395 patients with at least one primary care visit for knee OA. Patients had an average age of 68 years and were predominately white and female (Table 1). Analysis of data extracts was validated against chart review of 60 patients in the validation cohort. Adherence to the two NSAID QIs was conducted through analysis of data extracts in the expanded cohort of 335 patients.

**Table 1:** Demographics of the validation cohort and expanded cohort

Variable	Validation cohort (*n =* 60)	Expanded cohort (*n =* 335)	
Primary diagnosis code, *n* (%)			
Unilateral primary OA, left knee	23 (38%)	81 (24%)	
Bilateral primary OA of knee	14 (23%)	113 (34%)	
Unilateral primary OA, right knee	13 (22%)	108 (32%)	
Unilateral primary OA, unspecified knee	10 (17%)	33 (10%)	
Age, mean years (SD)	68 (10)	68 (10)	
Female sex, *n* (%)	38 (63%)	246 (73%)	
Race, *n* (%)			
White	43 (72%)	249 (74%)	
Black	8 (13%)	62 (19%)	
Other	9 (15%)	24 (7%)	

OA, osteoarthritis; SD, standard deviation

### Validation of data extract with chart review in the validation cohort (n = 60)

#### Validation of data elements

Compared to chart review, analysis of data extracts had high specificity and rarely resulted in a false positive for identifying concomitant medication use (Table 2). However, the sensitivity was limited for NSAIDs (73%). Data extract did not identify OTC ibuprofen in 4 patients, OTC naproxen in 2 patients, and an unspecified OTC NSAID in 1 patient, which were identified by reading clinic visit notes during chart review. Of 3 cases of PUD identified by chart review, the data extract only identified 1 case (sensitivity 33%). When all data elements of GI risk were evaluated together (age, medications, and PUD), data extract had a sensitivity of 98% and specificity of 100% compared to chart review. The one case of CKD identified by chart review was also identified by data extract (sensitivity 100%). Data extracts had a high specificity, rarely resulting in false positives, but had limited sensitivity for NSAIDs and PUD.

**Table 2:** Medication use from chart review versus data extract in validation cohort (*n =* 60)

Data element	Chart review ^a^	Data extract ^a^	Sensitivity ^b^	Specificity ^b^
Concomitant medication use				
NSAIDs	26 (43%)	19 (32%)	73% (52–88%)	100% (90–100%)
PPIs	19 (32%)	18 (30%)	95% (74–100%)	100% (91–100%)
Aspirin	16 (27%)	15 (25%)	94% (70–100%)	100% (92–100%)
Anticoagulants	5 (8%)	5 (8%)	100% (48–100%)	100% (94–100%)
Antiplatelets	6 (10%)	6 (10%)	100% (54–100%)	100% (93–100%)
Steroids	0 (0%)	0 (0%)	N/A	N/A
CKD stage G4 or G5	1 (2%)	1 (2%)	100% (3–100%)	100% (94–100%)
History of peptic ulcer disease	3^c^ (5%)	1 (2%)	33% (1–91%)	100% (94–100%)
Clinical interpretation of GI risk	47 (78%)	46 (77%)	98% (89–100%)	100% (75–100%)

CKD, chronic kidney disease; GI, gastrointestinal; N/A, not applicable; NSAIDs, nonsteroidal anti-inflammatory drugs; PPI, proton pump inhibitor

^a^ Data presented as *n* (%)

^b^ Data presented for data extract compared to reference standard of chart review with accompanying 95% confidence interval

^c^ Of the two patients with a history of peptic ulcer disease from chart review that were missed by data extract, one patient had another gastrointestinal risk factor (age > 65 years old) and was correctly categorized as having elevated gastrointestinal risk by analysis of data extract

#### Validation of adherence to QI #1 NSAID-PPI

Adherence to QI #1 NSAID-PPI was 82% (95% CI 70–90%; 49 of 60) using chart review and 83% (95% CI 71–91%; 50 of 60) using data extracts. Compared to chart review, analysis of data extracts had a sensitivity of 100% (95% CI 93–100%) and specificity of 91% (95% CI 59–100%). Analysis of data extracts using our approach was interpreted as being valid.

#### Validation of adherence to QI #2 NSAID-CKD

Adherence to QI #2 NSAID-CKD was 100% (60 of 60) using chart review and 100% (60 of 60) using data extract. Compared to chart review, analysis of data extracts had 100% accuracy. Sensitivity and specificity could not be calculated due to perfect agreement. Analysis of data extracts using our approach was interpreted as being valid.

### Evaluation in the expanded cohort (n = 335)

#### Adherence to QI #1 NSAID-PPI

The prevalence of concomitant medication use was 44% (*n =* 149) for NSAIDs, which were predominately meloxicam (*n =* 77), naproxen (*n =* 26), ibuprofen (*n =* 24), celecoxib (*n =* 21), and diclofenac (*n =* 10). Uncommonly used NSAIDs included etodolac (*n =* 4), nabumetone (*n =* 4), fenoprofen (*n =* 2), ketorolac (*n =* 1), indomethacin (*n =* 1), and piroxicam (*n =* 1). The prevalence of concomitant PPI use was 27% (*n =* 92).

Use of medications that elevate GI risk was common (43%, *n =* 145), consisting of aspirin (34%, *n =* 113), anticoagulants (7%, *n =* 25), antiplatelets (6%, *n =* 21), and steroids (3%, *n =* 9). 60% (*n =* 201) of patients were older than 65 years old. The prevalence of PUD was 1% (3 of 335) identified using diagnosis codes. Therefore, 73% (*n =* 245) of patients had at least one GI risk factor. When placed into mutually exclusive categories, 56% (*n =* 186) had no NSAID or NSAID was discontinued, 17% (*n =* 57) had NSAID but no GI risk, 6% (*n =* 21) had NSAID and GI risk with PPI, and 21% (*n =* 71) had NSAID with GI risk but no PPI. Therefore, adherence to QI #1 NSAID-PPI was 79% (95% CI, 74–83%).

#### Adherence to QI #2 NSAID-CKD

The prevalence of CKD G4/G5 was 2% (8 of 335) with 3 cases identified using both diagnosis codes and eGFR values, 4 cases identified using eGFR values only, and 1 case identified using diagnosis codes only. When placed into mutually exclusive categories, 56% (*n =* 186) had no NSAID, and 44% (*n =* 149) had NSAID but not CKD. Therefore, adherence to QI #2 NSAID-CKD was 100%.

## Discussion

The substantial use of NSAIDs (44%) in our cohort of patients with primary care visits for knee OA underscores the importance of optimizing safety of NSAID use to prevent GI or renal injury. Adherence to QI #1 NSAID-PPI was 79%, suggesting that many knee OA patients who take NSAIDs also have elevated GI risk and would benefit from the addition of a PPI. Future research is needed to develop and implement interventions to improve adherence to these QIs and to improve structured documentation of OTC NSAID use in the EHR. Our team identified two initial opportunities to assist PCPs with identifying patients who may benefit from a PPI using clinical decision support in the EHR. First, we propose development of EHR clinical decision support to display GI risk factors and presence or absence of PPI therapy in the NSAID prescription order composer in the primary care setting. Second, an automated EHR alert could suggest prescribing a PPI when a NSAID is newly prescribed or continued for a patient with elevated GI risk but without concomitant PPI therapy. Compared with a manual chart review, our team found the EHR extract had a sensitivity of 98% and specificity of 100% for identifying GI risk. This suggests that the EHR can reliably identify patients at risk with few false positives, which is an important consideration for minimizing alert fatigue [31]. Although we observed adherence to QI #2 NSAID-CKD was 100%, the EHR could be enhanced with a safety net automated alert that could display a warning message if an NSAID is newly prescribed or continued for a patient with an ICD-10-CM code for CKD stage G4 or G5 or who has a documented eGFR < 30 mL/min/1.73 m^2^ in the prior 12 months. The accuracy of NSAID documentation is an important consideration for EHR-based interventions. Our study found NSAIDs were documented as structured data on the medication list for 73% of visits when compared with a manual chart review. The sensitivity of the future EHR interventions could be increased by focusing on enhancing NSAID documentation.

### Prevalence of CKD

Analysis of ICD-10-CM codes had a high sensitivity (100%) and specificity (100%) for identifying CKD stage G4 or G5 compared to chart review. However, the prevalence of CKD stage G4 or G5 was rare (2%) in the expanded cohort. The prevalence of CKD stage G3 is likely much higher, and additional research should obtain consensus on which comorbid conditions or concomitant medications serve as relative contraindications for NSAID use among OA patients with CKD in the primary care setting. If consensus is obtained, this QI could be revised to include CKD stage G3.

### Prevalence of GI risk

The prevalence GI risk was high (73%) in the expanded cohort, predominately driven by an age of over 65 years old (60%) and concomitant use (43%) of aspirin, anticoagulants, antiplatelets, or steroids. Given the rare occurrence of PUD observed in both cohorts, the low sensitivity of ICD-10-CM codes for detecting PUD had minimal impact on assessment of GI risk. However, future efforts could identify opportunities to improve documentation of PUD in structured healthcare data.

### Limitations

Pharmacological classes were not provided in the data extract, and medications were manually categorized by a pharmacist investigator. The process of assigning pharmacological categories to each medication would need to be automated before scaling this analysis to a larger cohort or real-time monitoring. Based on analysis of the validation cohort, the use of OTC ibuprofen and OTC naproxen is likely underreported in the expanded cohort. Furthermore, some use of OTC NSAIDs may not be documented in EHR medication lists or clinic visit notes. However, the risk associated with intermittent OTC NSAIDs may be much lower than the risk associated with scheduled use of prescription NSAID therapy. The operational definition of QI #1 NSAID-PPI does not detect overprescribing of PPIs and cannot identify patients using PPIs for other indications. Adherence to QIs may differ at other health systems that use different EHRs or that care for patients with a different prevalence of GI risk and CKD. The sample size of the validation cohort was chosen based on feasibility and was not derived using power calculations for formal testing rules to establish statistical significance for validity testing. This analysis was limited to the first (index) primary care clinic visits for knee OA for each patient in 2019, and metrics of adherence may vary in an analysis of all encounters (index and non-index).

## Conclusion

Patients with primary care visits for knee OA commonly (44%) use NSAIDs. Among this cohort with a high prevalence of GI risk factors (73%), adherence to QI #1 NSAID-PPI was 79% suggesting an opportunity to improve the safety of NSAID use by adding a PPI to eligible patients during primary care visits for knee OA. Among this cohort with low prevalence of CKD stage G4 or G5, adherence QI #2 NSAID-CKD was 100%. Future research can use these operational definitions to evaluate the impact of implementing EHR enhancements that aim to improve safe NSAID prescribing among OA patients in the primary care setting.

### Electronic supplementary material

Below is the link to the electronic supplementary material.

Supplementary Material 1

## Data Availability

All data generated or analyzed during this study are included in this published article and its supplementary information files.

## Abbreviations

AKI: Acute kidney injury
CKD: Chronic kidney disease
eGFR: Estimated glomerular filtration rate
EHR: Electronic health record
GI: Gastrointestinal
ICD-10-CM: International classification of diseases, tenth revision, clinical modification
NSAID: Nonsteroidal anti-inflammatory drug
OA: Osteoarthritis
OTC: Over the counter
PCP: Primary care physician
PPI: Proton pump inhibitor
PUD: Peptic ulcer disease
QI: Quality indicator

## Declarations

### Prior presentation

This work was not previously presented before submission to the journal.
