# Supplementary material for: Specification, validation, and adherence of quality indicators to optimize the safe use of nonsteroidal anti-inflammatory drugs for knee osteoarthritis pain in the primary care setting
Source: BMC Musculoskelet Disord. 2023 Sep 27;24:761. doi: 10.1186/s12891-023-06904-x (PMC10523610; doi:10.1186/s12891-023-06904-x)
Supplement: Supplementary file 1 — Supplementary Material 1 [file 12891_2023_6904_MOESM1_ESM.docx]

**Additional File 1**

**Supplemental Table 1. Diagnosis codes for peptic ulcer disease**

| **ICD-10-CM code** | **ICD-10-CM code description** |
| --- | --- |
| K2210 | Ulcer of esophagus without bleeding |
| K2211 | Ulcer of esophagus with bleeding |
| K250 | Acute gastric ulcer with hemorrhage |
| K251 | Acute gastric ulcer with perforation |
| K252 | Acute gastric ulcer with both hemorrhage and perforation |
| K253 | Acute gastric ulcer without hemorrhage or perforation |
| K254 | Chronic or unspecified gastric ulcer with hemorrhage |
| K255 | Chronic or unspecified gastric ulcer with perforation |
| K256 | Chronic or unsp gastric ulcer w both hemorrhage and perf |
| K257 | Chronic gastric ulcer without hemorrhage or perforation |
| K259 | Gastric ulcer, unsp as acute or chronic, w/o hemor or perf |
| K260 | Acute duodenal ulcer with hemorrhage |
| K261 | Acute duodenal ulcer with perforation |
| K262 | Acute duodenal ulcer with both hemorrhage and perforation |
| K263 | Acute duodenal ulcer without hemorrhage or perforation |
| K264 | Chronic or unspecified duodenal ulcer with hemorrhage |
| K265 | Chronic or unspecified duodenal ulcer with perforation |
| K266 | Chronic or unsp duodenal ulcer w both hemorrhage and perf |
| K267 | Chronic duodenal ulcer without hemorrhage or perforation |
| K269 | Duodenal ulcer, unsp as acute or chronic, w/o hemor or perf |
| K270 | Acute peptic ulcer, site unspecified, with hemorrhage |
| K271 | Acute peptic ulcer, site unspecified, with perforation |
| K272 | Acute peptic ulcer, site unsp, w both hemorrhage and perf |
| K273 | Acute peptic ulcer, site unsp, w/o hemorrhage or perforation |
| K274 | Chronic or unsp peptic ulcer, site unsp, with hemorrhage |
| K275 | Chronic or unspecified peptic ulcer, site unspecified, with perforation |
| K276 | Chr or unsp peptic ulcer, site unsp, w both hemor and perf |
| K277 | Chronic peptic ulcer, site unsp, w/o hemorrhage or perf |
| K279 | Peptic ulc, site unsp, unsp as ac or chr, w/o hemor or perf |
| K280 | Acute gastrojejunal ulcer with hemorrhage |
| K281 | Acute gastrojejunal ulcer with perforation |
| K282 | Acute gastrojejunal ulcer w both hemorrhage and perforation |
| K283 | Acute gastrojejunal ulcer without hemorrhage or perforation |
| K284 | Chronic or unspecified gastrojejunal ulcer with hemorrhage |
| K285 | Chronic or unspecified gastrojejunal ulcer with perforation |
| K286 | Chronic or unsp gastrojejunal ulcer w both hemor and perf |
| K287 | Chronic gastrojejunal ulcer w/o hemorrhage or perforation |
| K289 | Gastrojejunal ulcer, unsp as acute or chr, w/o hemor or perf |
| K633 ^a^ | Ulcer of intestine |
| K9281 | Gastrointestinal mucositis (ulcerative) |

^a^ Although code K633 does not specify the location of the ulcer within the small intestine, it was included to maximize sensitivity.

ICD-10-CM, international classification of diseases, tenth revision, clinical modification

**Supplemental Table 2. Diagnosis codes for chronic kidney disease stages G4 or G5**

| **ICD-10-CM code** | **ICD-10-CM code description** |
| --- | --- |
| I120 | Hypertensive chronic kidney disease with stage 5 chronic kidney disease or end stage renal disease |
| I1311 | Hypertensive heart and chronic kidney disease without heart failure, with stage 5 chronic kidney disease, or end stage renal disease |
| I132 | Hypertensive heart and chronic kidney disease with heart failure and with stage 5 chronic kidney disease, or end stage renal disease |
| N184 | Chronic kidney disease, stage 4 (severe) |
| N185 | Chronic kidney disease, stage 5 |
| N186 | End stage renal disease |

ICD-10-CM, international classification of diseases, tenth revision, clinical modification

Codes for chronic kidney disease were excluded if the severity stage was not explicitly listed.
